# The Effects of Adjuvant Fermented Wheat Germ Extract on Cancer Cell Lines: A Systematic Review

**DOI:** 10.3390/nu10101546

**Published:** 2018-10-19

**Authors:** Khrystyna Zhurakivska, Giuseppe Troiano, Vito Carlo Alberto Caponio, Mario Dioguardi, Claudia Arena, Lorenzo Lo Muzio

**Affiliations:** Department of Clinical and Experimental Medicine, University of Foggia, 71122 Foggia, Italy; khrystyna.zhurakivska@unifg.it (K.Z.); giuseppe.troiano@unifg.it (G.T.); karlos_93@hotmail.it (V.C.A.C.); mario.dioguardi@unifg.it (M.D.); claudia.arena@unifg.it (C.A.)

**Keywords:** FWGE, AVEMAR, fermented wheat germ extract, nutraceuticals, cancer treatment

## Abstract

Fermented wheat germ extract (FWGE; trade name AVEMAR) is a natural compound derived from industrial fermentation of wheat germ. Its potential anticancer properties has emerged from recent studies. The aim of this systematic review is to summarize the data available in the scientific literature concerning the in vitro activity of FWGE on malignant cells. A systematic review of English articles in electronic databases has been performed. The primary outcomes of the review regarded types of cancer cell lines subjected to the investigation and the main results concerning cell viability, proliferation, and apoptosis observed within the studies. Sixteen articles were included in the final qualitative analysis. Various types of cancer cells treated with FWGE have been analyzed, showing mainly cytotoxic effects, alteration of the cell cycle, antiproliferative effects, and induction of apoptosis. FWGE can be a promising drug component in cancer treatment; however, further in vitro and in vivo studies are necessary to prove its effectiveness and safety in humans.

## 1. Introduction

Recently, more and more attention has been placed on the use of nutraceuticals as therapeutic agents for cancer prevention, as well as supplements to conventional therapy because of their promising effects and a low rate of toxicity [1,2,3]. A number of natural compounds have been found to inhibit one or more pathways that contribute to proliferation of cancer cells and metastatic processes [4]. The most investigated and promising compounds studied in the last years include: curcumin [5], resveratrol [6], and indole-3-carbinol [7], which are naturally present in some species of fruit and vegetables.

Fermented wheat germ extract (FWGE; trade name AVEMAR) is a product of industrial fermentation of wheat germ. Its production process is patented and is derived from the extraction of wheat germ and fermentation by *Saccharomyces cerevisiae*, followed by separation of the fermentation liquid, drying, and then granulation. As with other nutraceuticals, FWGE contains various molecules, but recent studies assume that the two quinones, 2-methoxy benzoquinone and 2, 6-dimethoxy benzoquinone, which are present in wheat germ as glucosides, are likely to be responsible for some of the biological properties of FWGE [8,9].

Quinones are cyclic organic compounds containing two carbonyl groups (C=O) linked to the cyclic structure of a conjugated system. Several anticancer compounds, e.g., Mitomycin C, Mitotraxan, Doxorubicin, and Daunorubicin, are quinone derivatives [10,11]. The anticancer characteristics of AVEMAR have been deeply investigated, and results have suggested its metabolic, antiproliferative, and antimetastatic effects [12,13,14].

The antimetabolic effects of FWGE on cancer cells seems to be due to a hypermetabolic state of the cancer cells and their upregulated utilization of glucose [15,16]. The antimetastatic effect of FWGE, besides the immune-reconstitution, may also be due to its cell adhesion inhibitory, cell proliferation inhibitory, apoptosis enhancing, and antioxidant characteristics, which have also been observed in some in vitro experiments [17]. The antiproliferative action has been investigated in in vitro and in vivo studies performed on various human cancer cell lines and animal models, and results have shown a reduction of tumor growth in a dose-dependent manner [8,17,18,19].

The antimetastatic effect of FWGE investigated in vitro and in vivo by several studies appears to be promising for FWGE to be used alone or in association with traditional anticancer agents [8,17].

The aim of this systematic review is to summarize the data available in the scientific literature concerning the in vitro activity of FWGE on malignant cells.

## 2. Materials and Methods

This systematic review was performed according to the Preferred Reporting Items for Systematic Reviews and Meta-Analyses (PRISMA) [20]. A systematic review of English articles in electronic databases (PubMed, Scopus, and Web of Science) was performed independently by two authors (KZ and GT) using search terms: (AVEMAR OR “wheat germ extract”) AND (cancer OR antitumoral OR anticancer). No restrictions were imposed on the study designs.

The criterion for inclusion in this systematic review was in vitro original studies on human tumor cell lines where the effects of FWGE have been evaluated. No restriction on publication dates was applied. No restriction of materials and methods was applied.

The exclusion criteria were letters to the editor, in vivo studies, and reviews. Articles and abstracts written in languages other than English were excluded.

A first selection was performed by reading the titles and the abstracts of the search results. After this round, duplicates resulting from the use of different databases were removed. The abstracts that seemed to meet the inclusion criteria were selected and the full texts were read. Once the full-text evaluation was performed, only studies meeting all inclusion criteria and considered eligible by both authors were included in the review. Disagreements between the authors were resolved through discussion.

Furthermore, the bibliographies of the included articles were examined in order to find other studies to include in this review.

The data concerning the type of the cells and the main evaluations performed on them were collected and the results are summarized in Table 1. No differentiation between methods was applied, and only the final results were considered.

The primary outcomes of the review regarded types of cancer cell lines subjected to the investigation and the main results concerning the cell viability, proliferation, and apoptosis observed in the studies. No quantification was made, but only significant results were considered and reported in the present review.

The secondary outcomes regarded other types of interventions and evaluations performed on the cells, their results in term of cellular metabolism, and enzymatic activity.

## 3. Results

A total of 56 titles and articles from PubMed, 53 from Scopus, and 52 from Web of Science were screened in the first round of the selection process. After duplicates were removed, 20 studies were identified as acceptable for full-text evaluation and their full texts were read. At the end of the selection process, 16 articles [13,18,19,21,22,23,24,25,26,27,28,29,30,31,32,33] were included in qualitative analysis, while four [9,17,35,36] were excluded for not complying with inclusion criteria.

**Figure 1:**
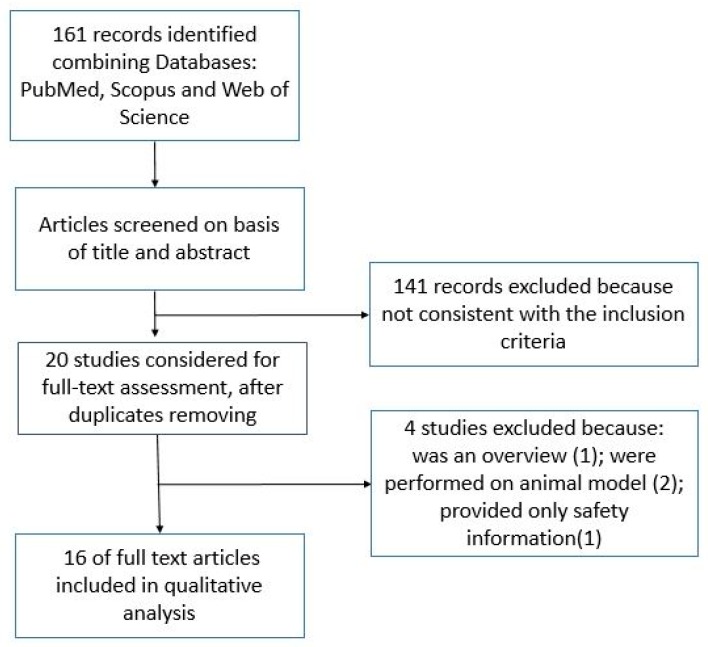
Flowchart of the selection process for the studies inclusion.

The flowchart in Figure 1 represents the selection process for the inclusion of studies. 

### 3.1. Characteristics of Included Studies and Primary Outcomes

All sixteen studies selected for the review were in-vitro studies published in English. The effects of FWGE were investigated on the following cell lines:

(1) Jurkat leukemic T cells were studied by three studies [18,21,32] and the treatment resulted in cytotoxic effects, alteration of the cell cycle, antiproliferative effects, and induction of apoptosis;
(2) Lymphoma cells were subjected to the treatment and the effects investigated by three studies [21,27,32] showed growth inhibition, induction of apoptosis, antiproliferation, and cytotoxic effects;
(3) Gastric cancer cell line experiments reported in three papers [22,25,26] found antiproliferative, cytotoxic, cytostatic, and growth-delay effects;
(4) Ovarian cancer cell lines, when subjected to treatment with AVEMAR, showed cytotoxic effects [23], antiproliferative activity [25], and suppression of cell proliferation [29];
(5) Breast cancer cell lines appeared to undergo cytotoxicity and apoptosis [24,25,26,32], while, in one study [33] investigating the combined administration of AVEMAR and cytostatics, the treatment with AVEMAR resulted in no increase or decrease of cell viability compared to untreated cells;
(6) When applied to colon cancer cell lines, FWGE showed antiproliferative activity [25], cytotoxicity, cytostasis, and induction of cell apoptosis [31];
(7) The treatment of hepatic cancer cells appeared to cause cytotoxicity, apoptosis [32], and an inhibition of proliferation [25,28];
(8) Other types of cell lines investigated in the included studies were prostate cancer cells, endocervical adenocarcinoma [22], cervical epidermoid carcinoma cells [25], testicular cancer cell lines [25], head and neck cancer [25], thyroid and pancreatic cancer cells [13], melanoma, hepatoma, glioblastoma, neuroblastoma [22], and oral squamous carcinoma cells [30]. In all cases, the effects of AVEMAR treatment provided results similar to those previously mentioned.

### 3.2. Secondary Outcomes

Additional results that emerged from the experiments include:

(1) Enzyme activities evaluation. In particular, FWGE was found to inhibit Glucose-6-phosphate dehydrogenase (G6PDH), Lactate dehydrogenase (LDH) and Hexokinase (HK) activity in Jurkat T-progeny leukemia cells [18]. The inhibition of ribonucleotide reductase (RR) activity in promyelocytic leukemia cells was established by Saiko et al. [19]. A suppression of the expression of matrix metalloproteinase-2 (MMP-2) and urokinase plasminogen activator (u-PA) was revealed in oral cancer cells (SCC-4) treated with Avemar [30];
(2) Presumable anti-angiogenic effects of FWGE on human cervical carcinoma (HeLa) and human lung adenocarcinoma (A549) cells through the inhibition of vascular endothelial growth factor (VEGF) and cyclooxygenase-2 (Cox-2) levels [22];
(3) Impaired glucose consumption and reduced production of lactic acid in some adenocarcinoma cell lines [26,34].

## 4. Discussion

Nutraceuticals are gaining importance in the prevention and treatment of different diseases. In particular, cancer treatment is a challenge that is still ongoing because of the difficulties presented by conventional drugs, which are often accompanied by serious side effects and unsatisfactory results.

FWGE is a nutraceutical that has been reported to possess unique “cancer-fighting” characteristics [13]. Its antiproliferative and cytotoxic activity, as well as its induction of apoptosis in human cancer cells, have been affirmed by several studies [18,21,24]. Some studies [18,26,34] investigated the metabolic changes in tumor cells in response to the treatment with FWGE and revealed important alterations in enzymes involved in direct glucose oxidation (G6PDH), non-oxidative glucose utilization (transketolase) toward nucleic acid synthesis, glycolysis (LDH), and glucose activation (HK). The inhibition of the key pathways of sugar metabolism and DNA-synthesis seems to contribute to the proliferation inhibiting capacity of FWGE. Saiko et al. [19] found that treatment with AVEMAR carried a direct enzyme attenuation of ribonucleotide reductase, which was demonstrated to be significantly up-regulated in tumor cells. In another study [21], some early biochemical events, such as tyrosine phosphorylation and the increase of intracellular Ca^2+^ concentration, occurred in response to the treatment and was associated with increased apoptosis of the tumor cells. Judson et al. [23] have also identified genes and molecular signaling pathways associated with FWGE activity on investigated cells and these pathways include hedgehog signaling, activin A signaling regulation, and regulation of GAP 1/Synthesis (G1/S) phases transition.

Moreover, some of the studies included in this review suggested that AVEMAR could potentially inhibit cancer cell migration and invasive capacities [30]. Such actions can be important especially for some types of tumors, such as the oral squamous cell carcinoma [37,38], for which the highest mortality is due to the ability of lymph node metastasis or metastasis to distant organs. Imir et al. [22] investigated the ability of FWGE to inhibit angiogenesis and obtained very encouraging results stating that AVEMAR exerts anti-angiogenic effects by inhibiting VEGF and Cox-2 gene expression. This mechanism of action has already been proposed for the anti-angiogenic activity of polyphenols and polyphenol-rich foods in “in vitro” and “in vivo” models of angiogenesis [39]. 

Another important finding was that the treatment with AVEMAR results in widespread apoptosis in lymphoid tumor cells, but it does not induce apoptosis of healthy resting mononuclear cells [21].

In some studies, the association of FWGE with conventional anticancer drugs has also been investigated in order to search for possible solutions to overcome the drug resistance that frequently occurs during anticancer chemotherapy and limit the side effects that traditional treatments entail. The results are promising for the addition of this natural compound to cisplatin chemotherapy of epithelial ovarian cancer cell lines [23,29] and hepatocellular carcinoma cells [28], to tamoxifen in the treatment of breast cancer cells [24], to docetaxel in ovarian carcinoma cells [29], to 5-Fluorouracil (5-FU) in colon cancer cells [25], and to the treatment of hepatocellular carcinoma cells [28]. Only one study [33] reported poor results for simultaneous use of AVEMAR and Dacarbazine, 5-fluorouracyl, or Adriblastina, where the addition of the nutraceutical did not increase nor decrease the viability of any of the cell cultures. However, the authors concluded that, based on the findings in the literature that have stated immunomodulatory and antimetastatic effects of AVEMAR [8,17,21,40,41], the latter may be administered with cytostatic drugs without increasing toxicity or decreasing the antiproliferative effect of the cytostatics. Concordant conclusions were reached by Yeend et al. [42] in their systematic review that considered clinical studies evaluating the adjunction of AVEMAR to conventional cancer treatments when compared to conventional cancer treatment alone.

It should be noted that, in some of the included studies [31,32], the nutraceutical was not purchased from the manufacturing company, but instead produced in the laboratory through the use of *Lactobacillus plantarum* dy-1 on fresh wheat germ. Barisone et al. [32] described a different way of obtaining the fermented wheat germ extract by using *Saccharomyces cerevisiae*. They also compared the in vitro activity of FWGE with that of AVEMAR and concluded that the killing activity was equivalent [32].

An important aspect to consider when experimenting with new principles is their safety. Although wheat germ is a commonly consumed food with no known adverse effects, the toxicity of AVEMAR has been investigated in various studies as summarized by Heimbach et al. [35]. Though this study reported that the use of AVEMAR pulvis would not be expected to cause adverse effects, the scientific literature on this is sparse. Further in vitro and in vivo studies are needed to assure its product safety as an ingredient in dietary supplements or as an anticancer drug.

## 5. Conclusions

The available data suggest that FWGE can be a promising compound that can be integrated into or improve the current treatment of cancer. However, further in vitro and in vivo studies are necessary to prove its effectiveness and safety in humans.

## Figures and Tables

**Table 1:** Main data of the included studies, reporting the first author, the year of publication, the title, the types of the cells subjected to the treatment, the investigation procedures performed, and the main results of the experiments.

Author	Year	Title	Cell Type	Investigations	Main Results	Secondary Outcomes
**Comin-Anduix et al. [18]**	2002	Fermented Wheat Germ Extract Inhibits Glycolysis/Pentose Cycle Enzymes and Induces Apoptosis through Poly (ADP-ribose) Polymerase Activation in Jurkat T-cell Leukemia Tumor Cells.	Jurkat T-cell Leukemia Tumor Cells.	Cell cycle analysis, cell viability assay, assessment of apoptosis.	Cytotoxic effects, alteration of the cell cycle, induction of apoptosis.	Cleavage of PARP, Transketolase, G6PDH, HK, LDH inhibition.
**Fajka-boja et al. [21]**	2002	Fermented wheat germ extract induces apoptosis and downregulation of major histocompatibility complex class I proteins in tumor T and B cell lines.	Jurkat leukemic T cells, Burkitt lymphoma B cell lines, myelo-monocytic cell line.	Detection of apoptotic cells, measurement of cell proliferation.	Induction of apoptosis, antiproliferative effect.	Elevation of intracellular Ca^2+^ concentration.
**Imir et al. [22]**	2018	Mechanism of the anti-angiogenic effect of AVEMAR on tumor cells.	NCI-N87 (gastric tubular adenocarcinoma), PC3 (prostate carcinoma), HeLa (adenocarcinoma) and A549 (lung adenocarcinoma)	Investigation of anti-angiogenic effects.	Inhibition of induced VEGF levels.	Inhibition of Cox-2 levels.
**Judson et al. [23]**	2012	Characterizing the efficacy of fermented wheat germ extract against ovarian cancer and defining the genomic basis of its activity.	Ovarian cancer cell lines.	Cell viability assays.	Cytotoxic effects, increase of cisplatin sensitivity.	
**Marcsek et al. [24]**	2004	The Efficacy of Tamoxifen in Estrogen Receptor–Positive Breast Cancer Cells Is Enhanced by a Medical Nutriment.	MCF-7 breast cancer cells.	Cytotoxic effects evaluation, detection of apoptosis and mitosis, evaluation of tamoxifen-combined treatment.	Cytotoxicity, induction of apoptosis.	
**Mueller et al. [25]**	2011	Promising cytotoxic activity profile of fermented wheat germ extract (Avemar^®^) in human cancer cell lines.	testicular cancer (H12.1, 2102EP, 1411HP, 1777NRpmet), colon cancer (HCT-8, HCT-15, HCT-116, HT-29, DLD-1, SW480, COLO205, COLO320DM), NSCLC (A549, A427, H322, H358), head and neck cancer (FADU, A253), cervical epidermoid carcinoma (A431), mammary adenocarcinoma (MCF-7, BT474), ovarian adenocarcinoma (A2780), gastric Cancer (M2), anaplastic thyroid cancer (8505C, SW1736), papillary thyroid cancer (BCPAP), follicular thyroid cancer (FTC133), melanoma, hepatoma (HepG2), glioblastoma (U87MG), neuroblastoma (SHSY5Y, SIMA).	Growth inhibition experiments, apoptosis evaluation.	Antiproliferative activity.	
**Otto et al. [26]**	2016	Antiproliferative and antimetabolic effects behind the anticancer property of fermented wheat germ extract.	Adenocarcinoma of the breast (MDA-MB-468) and (MDA-MB-231) and (BT-20), adenocarcinoma of the pancreas (ASPC-1) and (BxPC-3), adenocarcinoma of the stomach (23132/87), adenocarcinoma of the colon (HT-29) and (HRT-18), invasive breast ductal carcinoma (MCF-7).	Effects on cell growth, Cell cycle analysis.	Cytotoxic, antiproliferative and growth delay effects.	Depletion in cellular ATP and decrease in the NADH/NAD+ ratio. Impaired glucose consumption and significantly reduced production of lactic acid. Induction of autophagy in HRT-18 cells.
**Saiko et al. [19]**	2007	Avemar, a nontoxic fermented wheat germ extract, induces apoptosis and inhibits ribonucleotide reductase in human HL-60 promyelocytic leukemia cells.	Human HL-60 promyelocytic leukemia cells.	Apoptosis evaluation, cell cycle distribution analysis.	Induction of apoptosis, cell growth inhibition.	Decreasing of dNTPs, direct enzyme attenuation (ribonucleotide reductase; RR).
**Saiko et al. [27]**	2009	Avemar, a nontoxic fermented wheat germ extract, attenuates the growth of sensitive and 5-FdUrd/Ara-C cross-resistant H9 human lymphoma cells through induction of apoptosis.	Human lymphoma cells H9, 5-FdUrd/Ara-C cross-resistant H9 human lymphoma cell line.	Growth inhibition assay, apoptosis evaluation.	Growth inhibition, induction of apoptosis.	
**Tai et al. [28]**	2013	Fermented Wheat Germ Extract Induced Cell Death and Enhanced Cytotoxicity of Cisplatin and 5-Fluorouracil on Human Hepatocellular Carcinoma Cells.	Hepatocellular carcinoma (HCC) HepG2, Hep3B, and HepJ5 cells.	Cell viability Assay, evaluation of cisplatin and 5-fluorouracil combined treatment.	Antiproliferative activity, enhanced cytotoxicity of chemotherapeutic.	
**Wang et al. [29]**	2015	Preclinical Evaluation on the Tumor Suppression Efficiency and Combination Drug Effects of Fermented Wheat Germ Extract in Human Ovarian Carcinoma Cells.	SKOV-3 and ES-2 human ovarian carcinoma cells.	Cell viability evaluation, cell death markers analysis, evaluation of cisplatin- or docetaxel-combined treatment.	Suppression of cell proliferation, caspase-related apoptosis activation, increased cytotoxicity of cisplatin and docetaxel.	
**Yang et al. [30]**	2016	Inhibitory Effects of AVEMAR on Proliferation and Metastasis of Oral Cancer Cells.	Human oral squamous carcinoma SCC-4 cells.	Cell viability evaluation, cell apoptosis assay wound-healing migration assay, cell invasion assay.	Inhibition of cell viability, induction of cell apoptosis, suppression of migration and invasion capacity.	
**Zhang et al. [31]**	2015	Effect of Fermented Wheat Germ Extract with Lactobacillus plantarum dy-1 on HT-29 Cell Proliferation and Apoptosis.	Human HT-29 colon cancer cells.	Growth inhibition assay, assessment of apoptosis.	High antiproliferative effects, induction of cell apoptosis.	
**Barisone et al. [32]**	2017	A purified, fermented, extract of Triticum aestivum has lymphomacidal activity mediated via natural killer cell activation.	Lymphoma cells, T-cell leukemia (Jurkat), lung (H1650), breast (MCF-7) and hepatic (HepG2) cancer cell lines.	Cytotoxic activity assay, apoptosis and cell cycle.	Cytotoxic activity, apoptotic activity.	
**Szende et al. [33]**	2004	Effect of Simultaneous Administration of Avemar^®^ and Cytostatic Drugs on Viability of Cell Cultures, Growth of Experimental Tumors, and Survival of Tumor-Bearing Mice.	Human breast adenocarcinoma cell line (MCF-7), hepatocyte carcinoma (HepG2).	Cytotoxicity testing of Avemar associated with various cytostatic drugs (5 FU, Dacarbazine, Adriblastina).	Did not increase nor decrease cell viability.	
**Boros et al. [34]**	2001	Wheat Germ Extract Decreases Glucose Uptake and RNA Ribose Formation but Increases Fatty Acid Synthesis in MIA Pancreatic Adenocarcinoma Cells.	MIA pancreatic adenocarcinoma cells.	Evaluation of glucose utilization rates and lactate production.	Regulation of tumor cell proliferation.	Inhibitory effect on glucose consumption, little effect on lactate production.

PARP: poly ADP ribose polymerase, ATP: Adenosine triphosphate, G6PDH: Glucose-6-phosphate dehydrogenase, LDH: Lactate dehydrogenase, NADH: reduced Nicotinamide adenine dinucleotide; NAD+: oxidized nicotinamide adenine dinucleotide; HK: Hexokinase, 5-FdUrd/ara-C: 5-fluorodeoxyuridine/cytosine arabinoside; HeLa: human cervical carcinoma, RT-qPCR: Total RNA isolation and reverse transcription-quantitative polymerase chain reaction, dNTPs: deoxyribonucleoside triphosphates; Cox-2: cyclooxygenase-2; VEGF: vascular endothelial growth factor; dNTPS: deoxyribonucleoside triphosphates.
